# Tracking leading anti-*Candida* compounds in plant samples; *Plumbago europaea*

**Published:** 2018-06

**Authors:** Marzieh Sobhani, Mahdi Abbas-Mohammadi, Samad Nejad Ebrahimi, Atousa Aliahmadi

**Affiliations:** 1 Department of Phytochemistry and Essential Oils Technology, Factually of Pharmaceutical Chemistry, Pharmaceutical Sciences Branch, Islamic Azad University, Tehran, Iran; 2 Department of Phytochemistry, Medicinal Plants and Drugs Research Institute, Shahid Beheshti University, G.C., Evin, Tehran, Iran; 3 Department of Biology, Medicinal Plants and Drugs Research Institute, Shahid Beheshti University, G.C., Evin, Tehran, Iran

**Keywords:** Bio-autography, Plumbagin, *Candida albicans*, *Gardnerella vaginalis*

## Abstract

**Background and Objectives:**

Due to the importance of finding new and more effective antifungal and antibacterial compounds against invasive vaginitis strains, this study was conducted for fast screening of plant samples.

**Materials and Methods:**

Thirty Iranian plant samples were successively extracted by *n*-hexane, ethyl acetate and methanol to obtain a total of 90 extracts. Each extract was prepared in six concentrations and evaluated for antifungal activity via a micro-broth dilution method. Further phytochemical study of the aerial parts of *Plumbago europaea*, as the most promising source of anti-*Candida* compounds (with minimum inhibitory concentration of about 7μg/ml), was carried out and antifungal activity in the ethyl acetate extract was tracked using a combination of HPLC time-based fractionation and Thin Layer Chromatography-Bioautography via a bioassay-guided fractionation procedure. The compounds in the active region of the chromatogram were purified by a combination of column chromatography and preparative TLC, and then structure elucidation was achieved by 1D and 2D NMR, mass spectrometry and UV spectra.

**Results:**

Seven compounds were isolated and identified: (1) plumbagin, (2) isoplumbagin, (3) 5, 8-dihydroxy-2-methyl-[1, 4] naphthoquinone, (4) droserone, (5) 7-methyljuglone, (6) Isozeylanone, and (7) methylene-3, 3′-diplumbagin. Antimicrobial activity of the purified compounds were also evaluated against *C. albicans* (MIC values ranging from 2 to 2500 μM) and *Gardnerella vaginalis* (MIC values ranging from 20 to 2500 μM).

**Conclusion:**

These naphthoquinone compounds could be surveyed for finding new and more effective anti-vaginitis agents via drug design approaches.

## INTRODUCTION

The antibiotic era is going to end by finding new anti-infective chemical structures which are of global interest. Natural products (NPs) play an important role in drug discovery, as about 68% of antimicrobial agents are derived from NPs directly or indirectly (1). Plants are among the most promising sources of antimicrobial agents with a wide variety of chemical compounds (2). Application of different bioassay techniques like TLC-Bioautography (2, 3) and HPLC-based activity profiling could help scientists achieve new scaffolds from natural sources such as plants (4–6).

*Candida albicans* is one of the most important human pathogens that results in several infections such as esophageal or genital candidiasis and invasive illness which may be raised as nosocomial infections (3). Increasing the resistance of this pathogen to clinically achievable doses of antifungal drugs can highlight the importance of the search for new antifungal compounds.

*Plumbago europaea*, belonging to the Plumbaginaceae family, is the only species growing wild in Iran. This plant is also widespread in Europe, the Mediterranean region, North Africa and south west Asia (7). Previous study of some *Plumbago* species have shown good biological activity like antiviral, antitumor, leishmanicidial, trypanocidal, antimalarial, and insecticide properties (8).

In the present study, the *P. europaea* L. was selected as the best candidate of anti-*Candida* activity from a collection of 30 plant species (Table 1) for further phytochemical evaluation. Herein, we report the isolation, structure elucidation and anti-*Candida* activity of some compounds of the aerial parts of this plant. Also antibacterial activity of purified compounds was evaluated against a standard strain of *Gardnerella vaginalis* as an important bacterial agent of vaginal infections (9).

**Table 1.** Anti-*Candida* activity of Ethyl acetate extract of assessed plants (MIC and MBC values as mg/ml).

*Salvia hydrangea*	MIC	4	*Marubium perviflorum*	MIC	4	*Stachys sp.*	MIC	1
	MBC	>4		MBC	4		MBC	4

*Salvia limbata*	MIC	4	*Heracleum persicum*	MIC	0.5	*Glaucium elegans*	MIC	2
	MBC	>4		MBC	4		MBC	4

*Echium amoenum*	MIC	4	*Pistacia vera*	MIC	4	*Stachys annua*	MIC	4
	MBC	4		MBC	4		MBC	4

*Stachys byzantina*	MIC	4	*Rhus coriaria*	MIC	4	*Phelomis sp.*	MIC	2
	MBC	4		MBC	4		MBC	4

*Tanacetum balsamita*	MIC	4	*Pistacia atlantica*	MIC	4	*Marubium sp.*	MIC	4
	MBC	>4		MBC	4		MBC	4

*Alkanna trichophyla*	MIC	4	*Scorzonera latifolia*	MIC	4	*Heliotropium europaeum*	MIC	4
	MBC	4		MBC	4		MBC	>4

*Chaerophyllum macrospermum*	MIC	4	*Papaver bracteatum*	MIC	4	*Salvia sahendica*	MIC	4
	MBC	4		MBC	4		MBC	4

*Salvia leriifolia*	MIC	2	*Salvia multicaulis*	MIC	2	*Nepeta meyari*	MIC	4
	MBC	>4		MBC	4		MBC	4

*Salvia verticillata*	MIC	4	*Ziziphus jujube*	MIC	4	*Punica granatum*	MIC	4
	MBC	>4		MBC	>4		MBC	4

*Prangus ferolaceae*	MIC	4	*Plumbago europaea L.*	MIC	0.007	*Salvia hypoleuca*	MIC	1
	MBC	4		MBC	0.007		MBC	>4

## MATERIALS AND METHODS

### General experimental procedures.

NMR spectra were recorded on a Bruker AVANCE III 500 MHz spectrometer operating at 500.13 MHz for ^1^H. A 1-mm TXI microprobe with a z-gradient was used for 1H-detected experiments. Spectra were analyzed using Bruker Topspin 3.0 software. CDCl_3_ for NMR were purchased from Armar Chemicals. Solvents used for extraction, column chromatography and HPLC were of technical grade and were purified by distillation. Silica gel (70–230 mesh) was used for column chromatography and percolated silica gel F_254_ (20*20 cm) plates for TLC (both Merck). Detection was at 254 and 366 nm or by spraying with phosphor molybdic acid reagent and subsequently heating (120°C for 5 min). Analytical RP-HPLC using a SunFire^TM^ C18 column (3.5 μM, 10 mm × 150 mm; Waters) equipped with a pre-column (3 × 10 mm) was used for analytical evaluations. Semi preparative RP-HPLC using a SunFire^TM^ C18 column (5.0 μM, 10 mm × 150 mm; Waters) equipped with a pre-column (5.0 × 10 mm) was also used for the isolation process. Antifungal compounds were purchased from HiMedia (Mombay, India).

### Plant materials.

Aerial parts of all plants were collected from different regions of Iran from May to August 2015. Plant samples were identified by Dr. Ali Sonboli, and their voucher specimens were deposited in the Herbarium of Medicinal Plants and Drugs Research Institute of Shahid Beheshti University, Tehran, Iran.

### Preparation of plant extracts.

About 20 g of each powdered dried plant sample was extracted successively with *n*-hexane, ethyl acetate (EtOAc) and methanol (MeOH) (3 × 250 mL each) at room temperature. The extracts were concentrated under reduced pressure at 40°C using a Rotary evaporator instrument to obtain 90 solvent less extracts which were further used in an *in vitro* antimicrobial assay.

### Antimicrobial assays: antifungal screening assays.

The broth micro-dilution method was carried out according to the standard protocol recommended by CLSI (Clinical Laboratory Standard Institute) to determine the minimum concentration of each purified compounds required for inhibition (MIC) of visible growth of *C. albicans* ATCC10231 (11). In brief, two-fold serial dilutions of each extract were made (in a concentration range of 4-0.125 mg/mL for plant extracts and 1 to 2500 μM for purified compounds) in sterile micro-dilution trays containing RPMI pH 7 supplemented by 2% (w/v) Dextrose and MOPS (0.165 M). Dimethyl sulfoxide (DMSO) was used as solvent of extracts and purified compounds in final concentrations lower than 1% (v/v). Thereafter, a suspension of tested strain was prepared from freshly cultured yeast in sterile normal saline that were adjusted to turbidity of 0.5 McFarland standard. The suspension was further diluted (1:1000) by sterile mentioned broth medium just before addition to trays containing a serial dilution of each compound. MICs were recorded after 24 hrs incubation at 37°C. For determination of the minimum fungicidal concentration (MFC), 100 μL of each no growth wells in MIC determination experiments was cultured on Potato dextrose agar plates and MFC values were recorded after 24 hrs incubation of plating at 37°C. MFC values were recorded as the lowest concentrations which resulted in killing of all tested yeast cells. Nystatin, Amphotericin B and Fluconazole were also assessed as standard antifungal agents. All experiments were done in triplicates.

According to the obtained results of the antifungal assay, EtOAc extract of *P. europaea* with MIC and MBC value of 7 μg/mL was selected for further investigation and identification of its active compounds.

### Evaluation of anti-Gardnerella activity of purified compounds.

*In vitro* antibacterial activity of purified compounds was also assessed against *G. vaginalis* (clindamycin resistant clinical isolate with MIC and MBC>1180 μM∼500μg/mL). Determination of MIC was performed by the broth micro-dilution method as recommended by CLSI (Clinical Laboratory and Standard Institute) with some modifications (12). In brief, a serial dilution of each compound was made in a concentration range of 1 to 2500 μM in sterile 96 wells trays containing Mueller-Hinton broth medium. DMSO was used as solvent of extracts and purified compounds in final concentrations lower than 1% (v/v). Normal saline was used for preparation of inoculants having turbidity equal to 0.5 McFarland standards. The inoculants of the microbial strains were prepared from freshly cultured bacteria that were adjusted to 0.5 McFarland standard turbidity and were further diluted (1:100) using MHB medium just before adding to the serial diluted samples. Trays were incubated for 24 h at 37°C. MIC values were recorded as the lowest concentrations which could inhibit visible growth of microorganisms. Minimum bactericidal concentrations (MBCs) were determined by culturing of 100 μl of each no-growth well onto nutrient agar plates and incubation at suitable temperature. MBC values were recorded as the lowest concentration which resulted in killing of 99.9% of tested microorganism. Each experiment was done in triplicate and clindamycin was used as the standard antibacterial agent.

### Extraction of polar and non-polar compounds.

Dried samples of *P. europaea* (420 g) (with voucher number of MPH-2548) were powdered and extracted at room temperature with 6 L *n*-hexane, EtOAc and MeOH (3 × 48 h), respectively. After filtration, 3 extracts were dried using the rotary evaporator instrument (Heidolph, Germany) and kept at 4°C. The yields of extracts were as follow: 0.6% *n*-hexane, 2.9% EtOAc and 1.7% MeOH.

### Micro-fractionation of the ethyl acetate extract of *P. europaea* using RP-HPLC.

Aliquots of EtOAc extract (300 μl of 40 mg/ml solution in DMSO) were separated using semi-preparative RP-HPLC column (C18) with 0.1% formic acid in H_2_O (solvent A) and MeCN (solvent B) using the following gradient: 20% B to 100% B in 25 minutes, hold for 10 minutes and then reduced to 20% B in 5 minutes. The detection wavelength was 400 nm. The flow rate was 4.0 ml/min. Forty one-minute fractions were collected in test tubes and transferred to 96-well, deep well plates. The plates were dried by a MiniVap^™^ nitrogen evaporator and kept on the fridge.

### TLC-Bioautography assay.

Each dried micro-fraction obtained from HPLC experiment was dissolved in 200 μl of MeOH. 20 μl of each micro-fraction were spotted on TLC plate. After that, a suspension of freshly cultured *C. albicans* ATCC 10231 was made in molten Mueller Hinton agar (50°C) medium to final concentration of 1–2×10^7^ cfu/mL. Spotted TLC plate was covered with this suspension evenly and plate was incubated at 37°C for 24 hrs. An aqueous solution of MTT (2 mg/mL) was used for visualization of any inhibition of growth as illustrated elsewhere (10).

### Isolation of antimicrobial compounds of *P. europaea*.

The antimicrobial assay resulted in identification of the EtOAc extract as the best extract against *C. albicans*. Dried dark green residue of selected extract (12 g) was separated by an open column chromatography (70–230 μm, 3.5 × 50 cm, 150 g silica gel) with a gradient of n-hexane-EtOAc (98:2, 2 L; 90:10, 2 L; 85:15, 2 L; 80:10, 2 L; 75:25, 2 L; 70:30, 2 L; 65:35, 2 L; 60:40, 2 L; 55:45, 2 L; 50:50, 2 L; 45:55, 2 L; 35:65, 2 L; 25:75, 2 L; 15:85, 2 L; 5:95, 2 L and 0:100, 2 L), followed by increasing concentrations of MeOH (up to 100%) in EtOAc (90:10, 2 L; 75:25; 2 L; 55:45, 2 L; 25:75, 2 L; 0:100, 2 L) and continued by adding H_2_O in MeOH (90:10, 250 mL; 50:50, 500 mL). The effluents were combined to 18 fractions based on TLC patterns which were detected on TLC plates under UV (254 and 360 nm) or by heating after spraying with phosphomolybdic acid reagent. Fraction 2 [eluted with hexane-EtOAc (98:2)] resulted in a crude needle orange crystal as compound 1 (640 mg). During combination and evaporation of fraction 6, a pellet was obtained from compound 2 (2 mg) as a yellow crystal. Fraction 5 (39 mg) was subjected to a preparative TLC (10.0 × 10.0 cm) and washed with *n*-hexane-EtOAc (92:8) as the mobile phase to afford 10 bands (f 5_(1–10)_). From band 5_(3)_ a red amorphous solid obtained as compound 3 (3.4 mg). Fraction 3 (19 mg) revealed 3 spots on TLC analysis which were very near to each other in R_f_ values. So, a semi-preparative RP-HPLC was used for separation. The mobile phase was included 0.1% formic acid in water (solvent A) and 0.1% formic acid in acetonitrile (solvent B) using the following gradient: 5% B to 100% B in 30 minutes, hold for 5 min. The flow rate was 4.0 ml/min. The detection wavelength was 400 nm. Compounds 4 and 5 obtained from t_R_ = 19.2 min and t_R_ = 20.65 min, respectively. Fraction 7 [92 mg eluted with hexane-EtOAc (88:12)] was subjected to a preparative TLC (7 × 10 cm) and eluted with hexane-EtOAc (96:4) as the mobile phase to afford five sub-fractions (f 7_(1–5)_). Sub-fraction f7_(4)_ was further subjected to other preparative TLC analysis to afford the isolation of 1.6 mg of compound 6 and 1.4 mg of compound 7.

### Compound 1 (Plumbagin).

ESI-MS 189 [M+H]+ & 187 [M-H]-; UV: λmax 209, 245 and 420 nm; ^1^H NMR (500 MHz, CDCl_3_) δ 11.86 (s, 1H, OH), 7.54 (d, *J* = 5.4 Hz, 1H, H-6), 7.53 (d, *J* = 4.1 Hz, 1H, H-8), 7.17 (dd, *J* = 5.4, 4.1 Hz, 1H, H-7), 6.73 (d, *J* = 1.5 Hz, 1H, H-3), 2.14 (d, *J* = 1.5 Hz, 3H, H-11). ^13^C NMR (125 MHz, CDCl_3_) δ 190.3 (C-4), 184.1 (C-1), 160.8 (C-5), 149.1 (C-2), 136.1 (C-8), 135.3 (C-3), 131.8 (C-9), 123.9 (C-7), 118.9 (C-6), 115.0 (C-10), 15.6 (C-11).

### Compound 2 (isoplumbagin).

ESI-MS 189 [M+H]+; ^1^H NMR (500 MHz, Chloroform-d) δ 11.86 (s, 1H, OH), 7.54 (m, H-5), 7.50 (m, 1H, H-7), 7.16 (dd, *J* = 8.3, 1.1 Hz, 1H, H-6), 6.76 – 6.68 (d, *J* = 1.3 Hz, 1H, H-3), 2.11 (d, *J* = 1.3 Hz, 3H, H-11). ^13^C NMR (125 MHz, CDCl_3_) δ 185.5 (C-1), 184.6 (C-4), 161.1 (C-8), 149.6 (C-2), 134.8 (C-3), 135.5 (C-6), 132.1 (C-9), 123.5 (C-7), 118.6 (C-5), 115.2 (C-10), 16.3 (C-11).

### Docking analysis.

Molecular docking analysis to study binding affinity of compounds was carried out using Glide application using the Schrodinger package (2016-2). The CYP51 (Lanosterol 14-alpha demethylase) protein was taken from the PDB database (PDB code: 5ESE). The protein structure was prepared using protein preparation on Maestro 10.6, where all water molecules and co-crystalized ligands were removed. Partial atomic charges were assigned according to the OPLS3 force field. The structures were then subjected to impact minimization with a cut off RMSD of 0.3 Å. The grid box was centered at particular residues of the protein and was generated with Grid generation application. The molecular docking analysis was performed in glide. Finally, the obtained data were visualized using Maestro 10.6 platform.

## RESULTS

**Fig. 1.**
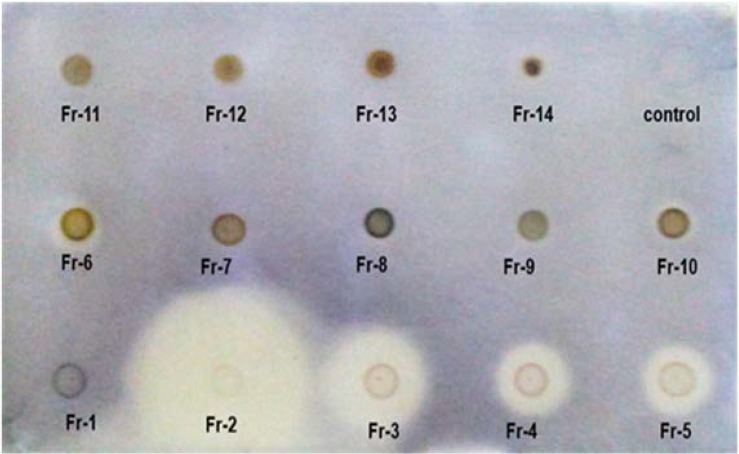
TLC-Bioautography of fractions 1–14 against *C. albicans*. 15 μg of each fraction was applied on silica plate as individual spots. The plate was covered by a suspension of yeast. MTT was used as growth indicator after incubation period. White (yellowish) zones show inhibition of growth and purple zones indicate growth. A 20 μL sample of MeOH was assessed as control.

According to the results of the medium-throughput screening of 90 extracts from 30 Iranian plants (MIC and MBC values of ethyl acetate extracts have been shown in Table 1), an EtOAc extract of the aerial parts of *P. europaea* revealed high antifungal activity against *C. albicans* with MIC and MFC values of 7 μg/mL. The selected extract was subjected to a phytochemical analysis to find its effective compounds for the antifungal activity. The fractionation of EtOAc extract using normal phase silica gel column chromatography resulted in obtaining 18 fractions. TLC-Bioautography of these fractions carried out using the dot-blot direct bio-autography method, showed that fractions 2–6 had antifungal activity, where the fraction 2 revealed very promising activity in comparing to the other fractions (Fig. 1).

**Fig. 2.**
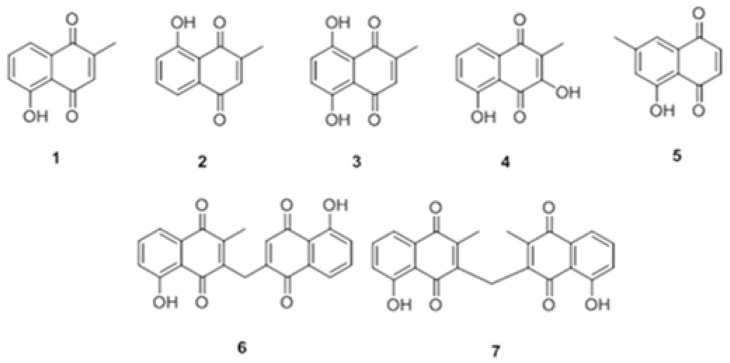
Chemical structure of isolated compounds (1–7) from *P. europaea*; the structures were elucidated by combination of UV-vis, Mass and NMR spectroscopy analysis.

Further chromatography of fractions in active regions resulted in isolation and identification of several compounds of *P. europae*a. The structure of isolated compounds was identified by combination of different spectroscopic methods such as 1D, 2D NMR (^1^H-^1^H COSY, HMQC, HMBC), and UV spectroscopy techniques. Moreover, mass spectroscopy analysis was used to prove identified structures through comparing their MS data with published data in the literature. The identified compounds (Fig. 2) belonged to naphthoquinone derivatives namely: (1) plumbagin, (2) isoplumbagin, (3) 5,8-dihydroxy-2-methyl-[1,4] naphthoquinone, (4) droserone, (5) 7-methyljuglone, (6) Isozeylanone, and (7) methylene-3,3′-diplumbagin.

The antimicrobial activity of isolated compounds against *G. vaginalis* and *C. albicans* were performed according to the CLSI guideline. The results are listed in Table 1. Among the isolated compounds, plumbagin (1); as the major component of the EtOAc extract, showed the highest activity with MIC and MBC values of 2 μM. This compound was also able to inhibit the growth of *G. vaginalis* tested strains as well (MIC and MBC values of 20 and 40 μM, respectively), while the standard antibiotic clindamycin had MIC and MBC values of >1180 μM. In addition, other compounds showed anti-microbial activity ranging from 1250 μM for compound 3 and 2500 μM for compounds 2, 6 and 7.

**Fig. 3.**
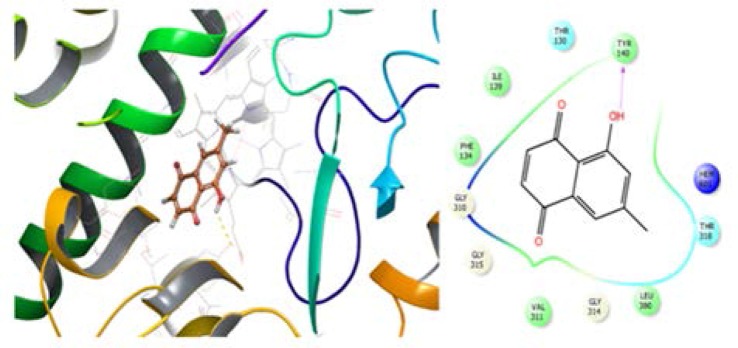
Interaction and docking model of active component with CYP51 (Lanosterol 14-alpha demethylase) in 3D (left) and 2D (right)

Lanosterol 14 α-demethylase (CYP51) is involved in the conversion of lanosterol to 4, 4-dimethylcholesta-8(9), 14, 24-trien-3β-ol in fungi and yeasts. This is commonly chosen as the biological target for designing antifungal drugs (13). Molecular docking of plumbagin showed a high affinity to the CYP51 protein with a docking score value of −5.473 kJ/mol. The activity of plumbagin was comparable with fluconazole as a positive control, which showed a docking score of −5.003 kJ/mol. The inspection of docking interaction between the receptor and compound 1 showed several hydrophobic interactions with ILE139, PHE134, VAL311, polar interactions with THR318, 130 and strong π-π stacking with PHE236 (Fig. 3). These interactions have significant similarity with those of fluconazole (as a native ligand) and receptor.

**Table 2.** Antimicrobial activity of *P. europaea* purified compounds (μM).

**Compound**	***Gardnerella vaginalis***		***Candida albicans ATCC10231***	

	**MIC^1^**	**MBC^2^**	**MIC^1^**	**MBC^3^**
plumbagin (1)	20	40	2	2
isoplumbagin (2)	2500	2500	2500	2500
5,8-dihydroxy-2-methyl-[1,4] naphthoquinone (3)	1250	2500	1250	1250
Isozeylanone (6)	2500	>2500	2500	>2500
methylene-3,3′-diplumbagin (7)	2500	>2500	2500	>2500
Nystatin	-	-	17	69
Amphotericin B	-	-	4	16
Fluconazole	-	-	1	2
Clindamycin	>1180	>1180	-	-

1. Minimum inhibitory concentration,

2. Minimum bactericidal concentration,

3. Minimum fungicidal concentration

## DISCUSSION

A bioassay guided fractionation of the EtOAc extract of *P. europaea* led to the isolation and identification of seven quinone derivatives showing good antimicrobial activity. This finding is in agreement with previous published data for similar compounds. Quinones, and especially 1, 4-naphthoquinone compounds, have been subjected to evaluation of their different biological activities such as decreasing of oxidative stress and as antimicrobial, antimalarial and anticancer agents (14–16). These compounds have great pharmacological interest which involves their link to aerobic metabolism and electron transferring mechanisms (17). In the literature, Plumbagin, a naturally occurring 1, 4-nathphtoquinone reported in *Plumbago, Drosera* and *Nepenthes* plant genera has shown several biological activities.

## CONCLUSION

This study provided a convenient and relatively fast approach for finding antimicrobial agents from plant samples. Also, results showed the effectiveness of plumbagin as an anti-vaginitis compound which inhibited two assessed important vaginal infective agents, as well. To the best of our knowledge, this is the first report on inhibitory effects of plumbagin on antibiotic resistant *G. vaginalis*. Regarding the complex nature of vaginal infections which are generally caused by different microorganisms (bacteria and yeasts), this compound or its derivatives could serve as promising therapeutic agents and can be surveyed as leading antimicrobial compounds in drug discovery and drug design studies.
